# MiR-29a Family as a Key Regulator of Skeletal Muscle Dysplasia in a Porcine Model of Intrauterine Growth Retardation

**DOI:** 10.3390/biom12091193

**Published:** 2022-08-28

**Authors:** Yan Zhu, Jianfeng Ma, Hongmei Pan, Mailin Gan, Linyuan Shen

**Affiliations:** 1 College of Life Science, China West Normal University, Nanchong 637009, China; 2 College of Animal Science and Technology, Sichuan Agricultural University, Chengdu 611130, China; 3 Farm Animal Genetic Resource Exploration and Innovation Key Laboratory of Sichuan Province, Sichuan Agricultural University, Chengdu 611130, China; 4 Chongqing Academy of Animal Science, Chongqing 402460, China

**Keywords:** skeletal muscle, intrauterine growth retardation, miR-29 family, IGF1, CCND1

## Abstract

MicroRNAs (miRNAs) play an essential role in many biological processes. In this study, miRNAs in the skeletal muscle of normal and intrauterine growth retardation (IUGR) neonatal piglets were identified by sequencing, and canonical miRNAs were functionally validated in vitro. A total of 403 miRNAs were identified in neonatal piglet skeletal muscle, among them 30 and 46 miRNAs were upregulated and downregulated in IUGR pigs, respectively. Upregulated miRNAs were mainly enriched in propanoate metabolism, endocytosis, beta-Alanine metabolism, gap junction, and tumor necrosis factor signaling pathway. Down-regulated miRNAs were mainly enriched in chemical carcinogenesis—receptor activation, endocytosis, MAPK signaling pathway, insulin resistance, and EGFR tyrosine kinase inhibitor resistance. Co-expression network analysis of umbilical cord blood and skeletal muscle miRNAs showed that the miR-29 family is an essential regulator of IUGR pigs. The dual-luciferase reporter system showed that IGF1 and CCND1 were target genes of the miR-29 family. Transfection of IUGR pig umbilical cord blood exosomes and miR-29a mimic significantly inhibited cell proliferation and promoted the expression of cellular protein degradation marker genes Fbxo32 and Trim63. In summary, these results enrich the regulatory network of miRNAs involved in skeletal muscle development in IUGR animals.

## 1. Introduction

Intrauterine growth retardation (IUGR) is a general term used for the developmental disorders of the fetus caused by maternal, fetal, placental abnormalities or other factors, mainly showing a decrease in birth weight and organ size of the newborn [1]. IUGR affects approximately 10–30% of newborns, and the proportion is higher in developing countries than in developed countries [2,3]. Clinical research shows that the IUGR is also a significant cause of perinatal infant death, having a detrimental effect on infants’ and young childrens’ growth and intellectual development. In terms of agricultural animals, IUGR is most common among multi-fetal mammals, especially pigs [4]. IUGR piglets generally have physiological defects such as overall slow growth, poor feed utilization, and poor meat quality [5]. In fetal blood circulation, blood preferentially supplies vital organs such as the brain and heart; however, the skin and muscles are at the end of the blood circulation and are frequently affected by IUGR [6]. Compared with normal-weight neonates, decreased skeletal muscle growth is typical in IUGR fetuses [7].

Skeletal muscle accounts for about 40% of body weight and is the primary executive tissue for motor function, and is widely involved in biological processes such as energy metabolism, endocrine functions, and immunity in the body [8]. Muscle mass is related to the number and diameter of muscle fibers, and numerous studies have shown that IUGR animals have fewer skeletal muscle fibers and smaller diameters [6,9]. The decrease in the number of secondary muscle fibers in IUGR animals is mainly related to environment and nutrition, which are also the main factors affecting the formation of IUGR [10]. The number of muscle fibers in mammalian skeletal muscle is fixed before birth [11]. Therefore, the loss of muscle mass caused by IUGR is not compensated after birth, and the effects will persist into adulthood.

microRNAs (miRNAs) are a class of non-coding single-stranded RNA molecules of approximately 22 nt length, involved in the post-transcriptional gene expression regulation [12]. Additionally, miRNAs are identified in various body fluids and participate in the mutual communication between tissues and organs [13]. In recent years, it has been reported that umbilical cord blood miRNA may be involved in angiogenesis [14], immunity [15], anti-inflammation [16], and other processes [17] exhibiting great prospects in disease treatment. Although umbilical cord blood stem cells have been used for clinical treatment in humans, the impact of cord blood miRNAs on fetal skeletal muscle development is yet to be explored.

The pig is an essential agricultural animal and an excellent model to study human diseases [18]. In this study, we identified miRNA expression signatures in normal and IUGR neonatal piglet skeletal muscle. Then, we combined them with IUGR pig umbilical cord blood miRNAs data to preliminarily analyze their effects on fetal skeletal muscle development.

## 2. Materials and Methods

### 2.1. Animals and Treatment

According to the farm data during the study period (1094 litter of pigs), the mean birth weight of piglets was 1.55 kg, and the standard deviation (SD) was 0.22. The piglets with birth weight lower than two SDs from the mean were defined as the IUGR group [19], and the piglets with birth weight within the ± 1 SD from the mean were defined as the normal group (Ming Xing Agriculture Science and Technology Development Co., LTD, Sichuan, China).

A total of 224 (IUGR, 75; Normal, 149) Yorkshire pigs were used for body weight and performance measurements. Six neonatal piglets were selected for the sample collection from three litters born on the same day (one IUGR pig and one normal pig per litter were selected based on the birth bodyweight). About 1 g of the longissimus dorsi muscle from the last rib was separated, then immediately placed in liquid nitrogen and stored at −80 °C for RNA extraction.

### 2.2. Small RNA Sequencing

Longissimus dorsi muscle from 3 normal piglets and 3 IUGR piglets were used for RNA sequencing. Total RNA was extracted from porcine skeletal muscle samples using the Trizol method. RNA concentration and quality were measured using the NanoDrop 2000 (Thermo, San Jose, CA, USA). Total RNA was subjected to small RNA library construction and sequencing (using the pooled samples). Small RNA sequencing was performed using the Illumina Nextseq 500 system. The sample collection, operation procedures, and processing and analysis of small RNA sequencing data were conducted according to a previously described protocol [20]. The raw reads produced in this study were deposited in the NCBI Sequence Read Archive (SRA), the records can be accessed by accession number PRJNA816268. The sequencing results of pig umbilical cord blood miRNAs were obtained from our previous report (GSE87111).

### 2.3. Prediction and Functional Annotation of Target Genes

The 3’UTR sequences of all pig genes were downloaded from Ensembl and compared with the seed sequences of miRNAs, and the target genes of miRNAs were determined with reference to the prediction results of Targetscan [21] (http://www.targetscan.org/vert_72/, accessed on 10 May 2022) and RNAhybrid [22] (https://bibiserv.cebitec.uni-bielefeld.de/rnahybrid, accessed on 12 May 2022). GO, and KEGG analyses were performed on predicted target genes [23].

### 2.4. Real-Time Quantitative PCR

Total RNA from the cells and tissues was extracted using TRIzol reagent (TaKaRa, Dalian, China). Reverse transcription of mRNA and miRNA was conducted according to the kit instructions (TaKaRa, Dalian, China). The primer sequences of IGF1 (insulin-like growth factor 1), CCNB1 (cyclin B1), CCND1 (cyclin D1), CDK4 (cyclin-dependent kinase 4), Fbxo32 (F-box protein 32) and Trim63 (tripartite motif-containing 63) were shown in Table S1.

### 2.5. Cell Culture and Transfection

Primary porcine skeletal muscle cells were obtained from a 2-day-old female DLY piglet. Primary porcine skeletal muscle cells were cultured at 37 °C and 5% CO_2_. The detailed protocol for preparing pig umbilical cord blood exosomes in advance is available in our previous report. miR-29a mimic, miR-29a inhibitor, and cord blood exosomes were transfected into cells using Lipo3000 (Invitrogen, Guangzhou, China) as a transfection reagent. Pig primary skeletal muscle cell-related experiments were transfected at approximately 30% cell density, focusing on cell proliferation.

### 2.6. Luciferase Reporter Assay

The IGF1 and CCND1 3’UTR sequence containing the miR-29 family binding site was inserted into the psiCHECKTM-2 vector, then the psiCHECKTM-2 vector and miR-29a mimic or negative control were co-transfected into HeLa cells using Lip3000. Firefly and renilla fluorescence were measured using Dual-Glo Luciferase Assay System (Promega, Madison, WI, USA) [24].

### 2.7. Statistical Analysis

All quantitative results were summarized using mean ± SD (standard deviation). Statistical analyses were conducted using SPSS 20.0 software (IBM, Almond, NY, USA). The differences between the groups were analyzed using Student’s t-test. The differences between the means were considered statistically significant for the *p*-value < 0.05.

## 3. Results

### 3.1. Skeletal Muscle Characteristics in Pigs with Intrauterine Growth Retardation

**Figure 1:**
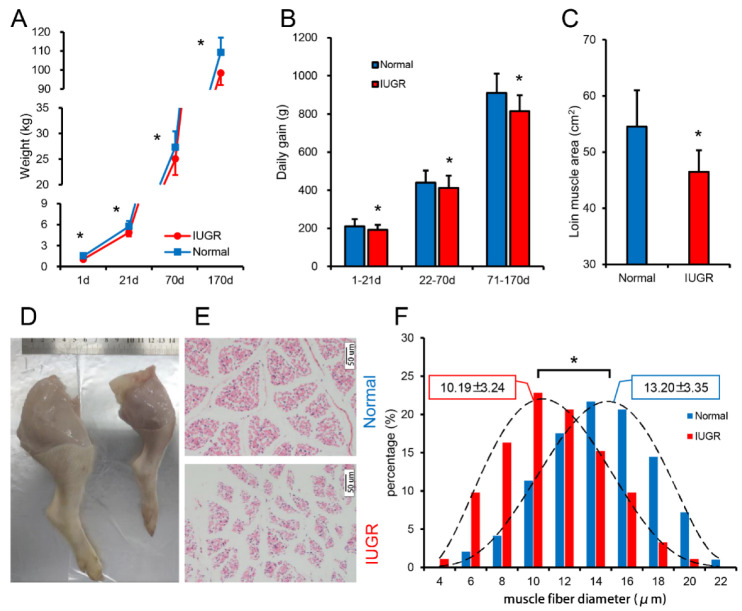
IUGR pigs with skeletal muscle dysplasia. (**A**) Birth to adult weight of normal and IUGR pigs; (**B**) Daily weight gain of normal and IUGR pigs at different stages; (**C**) Loin muscle area of 170–day–old normal and IUGR pigs, measurement using B-mode ultrasound; (**D**) Photographs of the legs of newborn normal and IUGR pigs; (**E**,**F**) HE (Hematoxylin and eosin) staining of longissimus dorsi muscle and muscle fiber diameter analysis. (**A**–**C**): Normal group, N = 149; IUGR group, N = 75; (**D**–**F**): N = 3. * *p*–value ≤ 0.05.

The body weight of IUGR pigs was significantly lower than that of normal pigs at birth (1-day), weaning (21-days), finishing (70-days), and market (170-days) ages (Figure 1A). The daily gain of IUGR pigs was also significantly lower than that of normal pigs in the lactation (1–21-days), nursery (22–70-days), and finishing (71–170-days) stages (Figure 1B). In vivo, B-ultrasound measurement showed that the loin muscle area of IUGR pigs was significantly lower than that of normal pigs (Figure 1C); these results suggest that the effects of IUGR are long-term, lasting into adulthood. Furthermore, we found that the muscle yield of newborn IUGR pigs was decreased, and the diameter of muscle fibers was significantly lower than that of normal pigs (Figure 1D–F).

### 3.2. Characterization of miRNAs in Normal and IUGR Pig Skeletal Muscle

**Figure 2:**
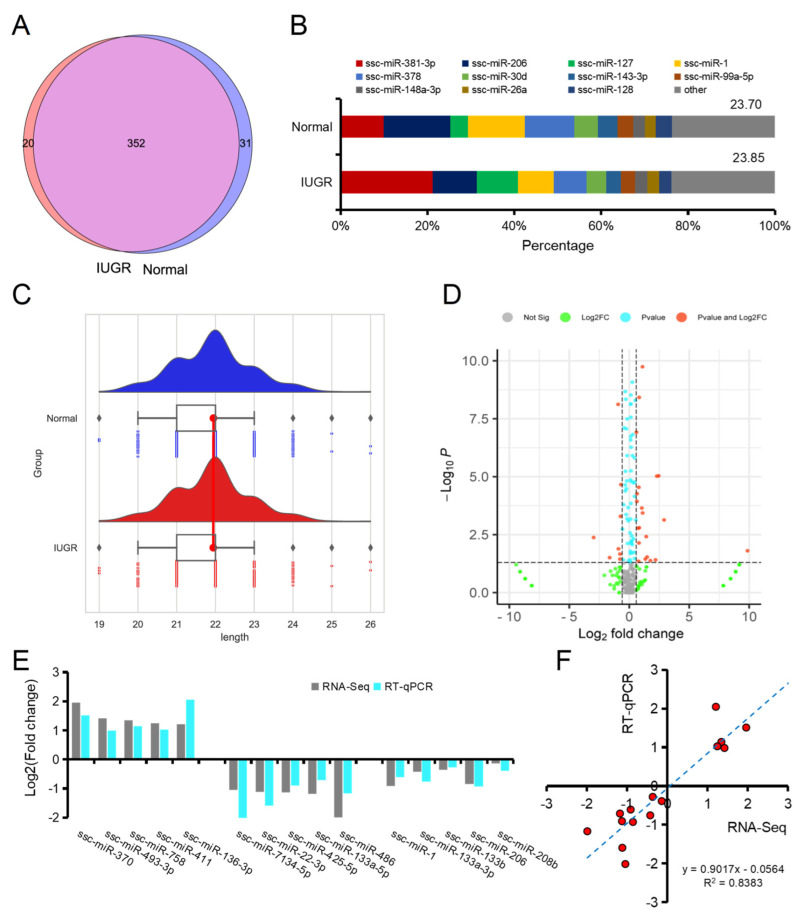
Expression characteristics of miRNAs in porcine skeletal muscle. (**A**) Species characterization of miRNAs in normal and IUGR pig skeletal muscle; (**B**) Top 10 miRNAs expressed in normal and IUGR pig skeletal muscle; (**C**) Length distribution of miRNAs in normal and IUGR pig skeletal muscle; (**D**) Volcano plot of differential miRNAs in normal and IUGR pig skeletal muscle; (**E**) RT-qPCR validation of partial sequencing results; (**F**) Correlation analysis between sequencing results and RT-qPCR results.

In this study, a total of 403 miRNAs were identified in the skeletal muscle of neonatal piglets, of which 31 were expressed explicitly in normal pigs, and 20 were expressed explicitly in IUGR pigs (Figure 2A). The most frequently expressed miRNA in the normal group was miR-206, and the top 10 miRNAs accounted for 76.30%, while in the IUGR group, the most frequently expressed miRNA was miR-381-3p, and the top 10 miRNAs accounted for 76.15% (Figure 2B). The length distribution of the two groups of miRNAs was similar, and the highest proportion was 22 nt (Figure 2C). Difference analysis showed that 30 miRNAs were upregulated, and 46 miRNAs were downregulated in the IUGR group (Figure 2D). RT-qPCR was used to verify the ten differentially expressed genes and the five myogenic miRNAs. We found that the trends were consistent, and the sequencing results were strongly positively correlated with RT-qPCR results (Figure 2E,F).

### 3.3. Functional Enrichment Analysis of Differentially Expressed miRNAs

**Figure 3:**
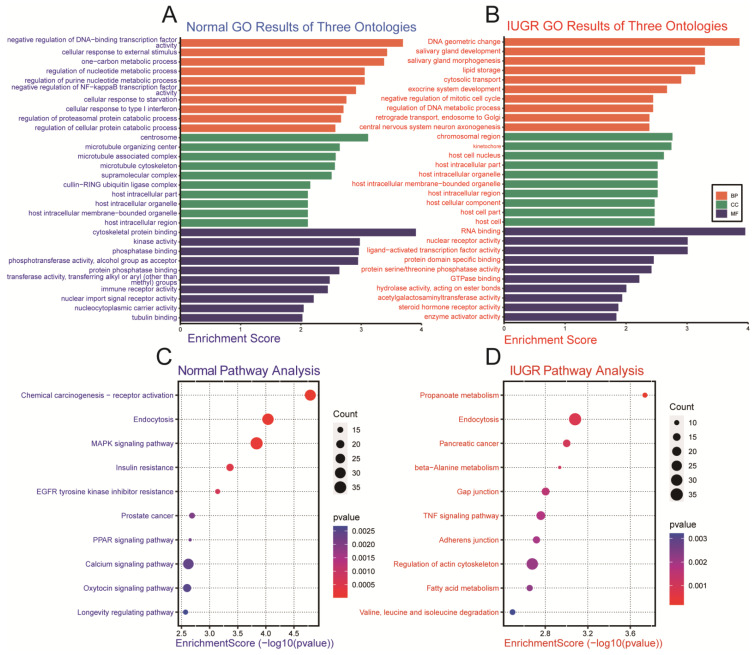
Functional enrichment analysis of differential miRNA in normal and IUGR pigs. (**A**) GO results of highly expressed miRNAs in the normal group; (**B**) GO results of highly expressed miRNAs in the IUGR group; (**C**) KEGG pathway analysis of highly expressed miRNAs in the normal group; (**D**) KEGG pathway analysis of highly expressed miRNAs in the IUGR group.

The 3’UTRs of all pig genes downloaded from the Ensembl genome browser (https://asia.ensembl.org/index.html, accessed on 25 July 2022) were compared with the seed sequences of differentially expressed miRNAs and the target genes were determined with reference to the RNAhybrid results (https://bibiserv.cebitec.uni-bielefeld.de/rnahybrid, accessed on 25 July 2022). GO (Gene Ontology) enrichment and KEGG (Kyoto Encyclopedia of Genes and Genomes) pathway analyses were performed on these target genes. GO analysis showed that the highly expressed miRNAs in the normal group were mainly annotated to metabolic processes, while the highly expressed miRNAs in the IUGR group were mainly annotated to developmental and cell cycle processes (Figure 3A,B, Table S2). KEGG analysis showed that the highly expressed miRNAs in the normal group were primarily enriched in chemical carcinogenesis: receptor activation, endocytosis, MAPK signaling pathway, insulin resistance, and EGFR tyrosine kinase inhibitor resistance signaling pathways. Further, the highly expressed miRNAs in the IUGR group were primarily enriched in Propanoate metabolism, endocytosis, beta-Alanine metabolism, gap junction, and TNF signaling pathway (Figure 3C,D).

### 3.4. Cord Blood and Skeletal Muscle miRNAs Regulatory Network in Normal and IUGR Pigs

**Figure 4:**
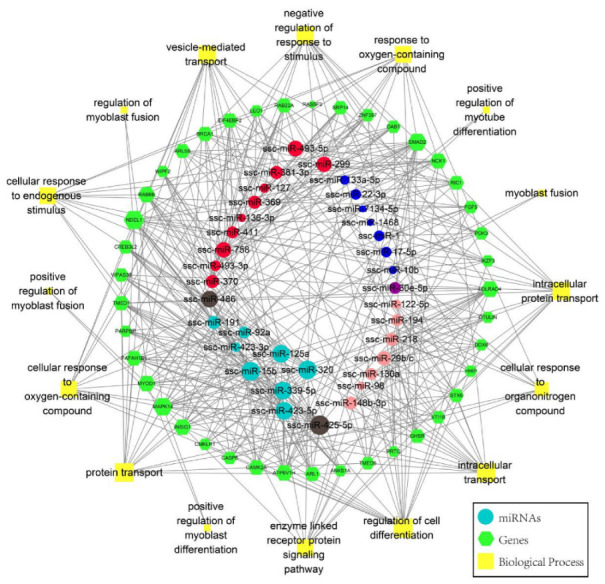
Expression network of differential miRNAs in neonatal pig skeletal muscle and umbilical cord blood. Red is the highly expressed miRNAs in IUGR pig skeletal muscle (IUGR-M group), blue is the highly expressed miRNAs in normal pig skeletal muscle (Normal-M group), pink is the highly expressed miRNAs in IUGR pig umbilical cord blood (IUGR-B group), light Blue is the highly expressed miRNAs in normal pig umbilical cord blood (Normal-B group), gray is the co-highly expressed miRNAs of IUGR-M group and Normal-B group, purple is the co-highly expressed miRNAs of Normal-M group and IUGR-B group.

**Figure 5:**
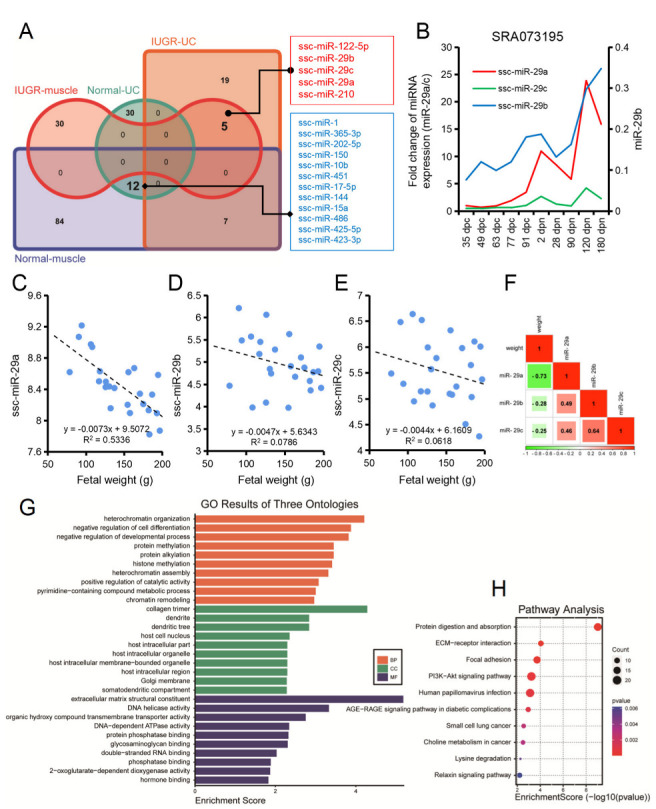
miR-29 family is central regulators of IUGR pig development. (**A**) Venn plots showing miRNA profiles in cord blood and skeletal muscle of normal and IUGR pigs; (**B**) Variation patterns of the miR-29 family during porcine skeletal muscle development; (**C**–**F**) Correlation analysis of relative expression levels of skeletal muscle miR-29 family with pig embryo weight; miR-29a (**C**), miR-29b (**D**), miR-29c (**E**), correlations among miRNAs within the miR-29 family (**F**). Data from GSE169093; (**G**) GO analysis results of common target genes of the miR-29 family; (**H**) KEGG analysis results of common target genes of the miR-29 family.

Differential miRNAs in umbilical cord blood and skeletal muscle of normal and IUGR pigs were analyzed, and 12.77% of the differently expressed miRNAs were found to have an intersection. The regulatory network analysis of miRNAs and their target genes showed that these differential miRNAs were mainly involved in the regulation of intracellular transport and were also related to the process of myogenesis (Figure 4). Twelve miRNAs were highly expressed in the normal group, and five miRNAs were highly expressed in the IUGR group (Figure 5A and Table S3). Specifically, among the highly expressed miRNAs in the IUGR group, miR-29a, miR-29b, and miR-29c belonged to the miR-29 family. According to the public data analysis, the miR-29 family had a similar expression pattern in different developmental stages of porcine skeletal muscle, which increased with age (Figure 5B). Correlation analysis showed that miR-29a, miR-29b, and miR-29c were negatively correlated with embryo weight to a certain extent, and the three miRNAs were positively correlated with each other (Figure 5C–F). According to GO and KEGG analyses of the target genes of the miR-29 family, the miR-29 family was specifically annotated to protein modification-related processes and was primarily enriched in protein digestion and absorption, ECM-receptor interaction, focal adhesion, PI3K-Akt signaling pathway, and AGE-RAGE signaling pathway in diabetic complications (Figure 5G,H).

### 3.5. IGF1 and CCND1 as Common Target Genes of the miR-29 Family

**Figure 6:**
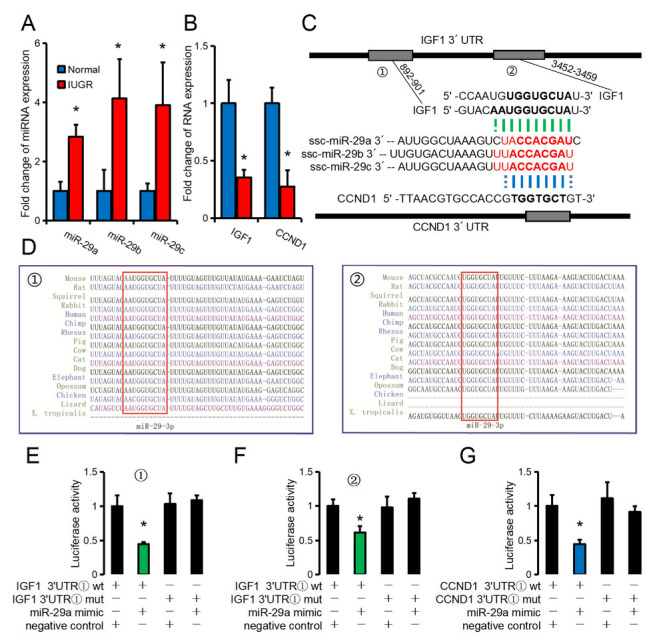
IGF1 and CCND1 as common target genes of miR-29a. (**A**) Relative expression of the miR-29 family in skeletal muscle of neonatal piglets; (**B**) Relative expression of IGF1 and CCND1 in skeletal muscle of neonatal piglets; (**C**) Potential binding sites of the miR-29 family to the 3’UTR of IGF1 and CCND1; (**D**) TargetScan results showed the conservation of potential binding sites of the miR-29 family to the 3’ UTR of IGF; (**E**,**F**) The dual-luciferase reporter system showed the binding relationship of miR-29a to the ① (**E**) and ② (**F**) of the 3’UTR of IGF1 and to the 3’UTR of CCND1 (**G**). N = 3. * *p*-value < 0.05.

miR-29a, miR-29b, and miR-29c were significantly higher in the skeletal muscle of IUGR pigs than in normal pigs; however, their potential target genes, IGF1 and CCND1, were significantly lower in the IUGR group than in the normal group (Figure 6A,B). The 3’UTR of CCND1 has a potential binding site for the miR-29 family (Figure 6C). The 3’UTR of IGF1 has two potential sites with the miR-29 family and is conserved in multiple species (Figure 6C,D). As miR-29a has the highest expression in skeletal muscle and has the best correlation with fetal weight (Figure 5B,C), we selected miR-29a for verification in vitro. The dual-luciferase reporter system confirmed the binding relationship between miR-29a and the 3’UTR of IGF1 and CCND1 (Figure 6E–G).

### 3.6. MiR-29 Family Is Involved in the Regulation of Umbilical Cord Blood miRNAs on Skeletal Muscle Development

**Figure 7:**
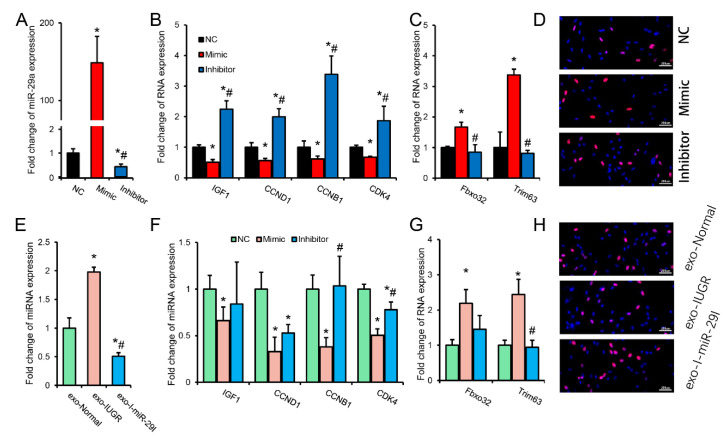
miR-29a is involved in the regulation of umbilical cord blood miRNAs in skeletal muscle cells in vitro. (**A**) The relative expression of miR-29a in pig primary skeletal muscle cells after transfection with miR-29a mimic or miR-29a inhibitor; (**B**) The relative expression of IGF1, CCND1 CCNB1, and CDK4 in pig primary skeletal muscle cells after transfection with miR-29a mimic or miR-29a inhibitor; (**C**) The relative expression of Fbxo32 and Trim63 in pig primary skeletal muscle cells after transfection with miR-29a mimic or miR-29a inhibitor; (**D**) EdU staining results of porcine primary skeletal muscle cells after transfection with miR-29a mimic or miR-29a inhibitor; (**E**) The relative expression of miR-29a in porcine primary skeletal muscle cells after co-treatment with normal porcine umbilical cord blood exosomes (exo-Normal) or IUGR porcine umbilical cord blood exosomes (exo-IUGR) and miR-29a inhibitor (exo-I-miR-29I); (**F**,**G**) The relative expression of IGF1, CCND1 CCNB1, CDK4, Fbxo32, and Trim63 in porcine primary skeletal muscle cells after co-treatment with exo-Normal or exo-IUGR and exo-I-miR-29I; (**H**) EdU staining results of porcine primary skeletal muscle cells after co-treatment with exo-Normal or exo-IUGR and exo-I-miR-29I. N = 3. * *p*-value < 0.05 vs. *NC,* # *p*-value < 0.05 vs. Mimic.

miR-29a was successfully overexpressed or inhibited in porcine primary skeletal muscle cells by transfection of miR-29a mimic or inhibitor (Figure 7A). Overexpression of miR-29a in porcine primary skeletal muscle cells significantly inhibited the expression of IGF1, CCND1, CCNB1, and CDK4 but significantly promoted the expression of Fbxo32 and Trim63 (Figure 7B,C). Although inhibiting the expression of miR-29a significantly promoted the expression of IGF1, CCND1, CCNB1, and CDK4, it had no significant effect on Fbxo32 and Trim63 (Figure 7B,C). Similarly, Edu test results showed that overexpression of miR-29a inhibited cell proliferation while inhibiting miR-29a promoted cell proliferation (Figure 7D). Since the miR-29 family was highly expressed in IUGR pig umbilical cord blood (Figure 5A), this study further explored the effect of normal and IUGR pig umbilical cord blood exosomes on porcine skeletal muscle primary cells. Incubation of IUGR porcine umbilical cord blood exosomes in porcine primary skeletal muscle cells significantly increased the expression of miR-29a compared with incubation of normal porcine umbilical cord blood exosomes. Further, co-transfection of miR-29a inhibitor significantly downregulated miR-29a expression (Figure 7E). Meanwhile, incubation of IUGR cord blood exosomes resulted in a significant decrease in the expression levels of IGF1, CCND1, CCNB1, and CDK4, and a significant upregulation of Fbxo32 and Trim63 in primary skeletal muscle cells of pigs (Figure 7F,G). Co-transfection of miR-29a inhibitor alleviated the adverse effects caused by incubation of IUGR pig umbilical cord blood partially (Figure 7F,G). The EdU proliferation test showed that incubation of IUGR pig umbilical cord blood exosomes inhibited the proliferation of porcine primary skeletal muscle cells compared with incubation of normal pig umbilical cord blood exosomes. Further, the addition of miR-29a inhibitor could partially recover from incubation of IUGR pig umbilical cord inhibition of proliferation by blood exosomes (Figure 7H); these results suggest that miR-29a is involved in the regulation of skeletal muscle development through IUGR pig umbilical cord blood miRNAs.

## 4. Discussion

IUGR is extremely common in mammals and an important adverse factor affecting human health and restricting livestock production [25]. About 30 million babies worldwide suffer from IUGR each year, and the incidence of IUGR in farm animals is around 10% [26]. The skeletal muscle is the largest organ in the body and one of the most severely affected by IUGR. Numerous studies have reported a positive relationship between birth weight and muscle mass, and the effects of IUGR on muscle persist into adulthood and are associated with a higher risk of metabolic diseases. miRNAs are widely present in body fluids and various organs and serve as communication mediators between tissues. Many studies have reported that miRNAs are essential regulators of animal skeletal muscle development and metabolism, but little is known about their effects on skeletal muscle development in IUGR animals [19]. In this study, we used pig as an animal model to analyze the characteristics of miRNAs in the skeletal muscle of newborn IUGR piglets. We found that the miR-29 family might be the key miRNAs involved in regulating fetal skeletal muscle development via umbilical cord blood miRNAs.

### 4.1. Characterization of miRNAs in Skeletal Muscle of Normal and IUGR Neonatal Piglets

Expression characteristics of miRNA differ in different tissues [27]. Interestingly, the expression of a few miRNAs accounted for most of the total miRNA expression [28]. In this study, we found that 9 of the top 10 miRNAs expressed in the normal and IUGR pig skeletal muscles were the same, and the top 10 miRNAs accounted for more than 75% of the expressions in skeletal muscle which was consistent with previous reports [29]. Among the co-highly expressed miRNAs, miR-1 and miR-206 are important myogenic miRNAs [30], while miR-378 [31], miR-381-3p [32], miR-30d [33], miR-143-3p [34], miR-127 [35], miR-99a-5p [36] and miR-148a-3p [37] were also reported to be involved in skeletal muscle development. Thus, these miRNAs are highly enriched in skeletal muscle and play an essential role in maintaining the normal function of skeletal muscle. The expression of miR-381-3p was the highest in IUGR pig skeletal muscle, accounting for 21.17% (normal: 9.86%), while the expression of miR-206 in normal pig skeletal muscle was the highest, accounting for 15.45% (IUGR: 10.13%). Previous studies have reported that miR-381-3p may be involved in the autolysis of skeletal muscle in the early stage of death [38]; these results suggest that miR-381 may play an important role in regulating skeletal muscle homeostasis in IUGR animals.

In this study, 30 miRNAs were upregulated in IUGR pig skeletal muscle, while 46 miRNAs were downregulated. RT-qPCR was performed on the top 100 miRNAs and myogenic miRNAs [39] with the highest expression levels and the largest fold difference. The sequencing results were highly correlated (R2: 0.84) with the RT-qPCR results, indicating the reliability of the sequencing results. Further, among the upregulated miRNAs in IUGR pig skeletal muscle, miR-411 was also reported to be upregulated in Facioscapulohumeral muscular dystrophy (FSHD) and inhibited myogenic factor expression [40]. Among the down-regulated miRNAs in IUGR pig skeletal muscle, miR-486 [41], miR-22-3p [42], and miR-133a-5p [43] were reported to be involved in skeletal muscle development; however, the role of these miRNAs in skeletal muscle development in IUGR animals remains unclear.

GO analysis showed that the highly expressed miRNAs in the normal group were mainly annotated to metabolic processes (one-carbon metabolic process, regulation of nucleotide metabolic process, and regulation of purine nucleotide metabolic process). In contrast, the highly expressed miRNAs in the IUGR group were mainly annotated to developmental (salivary gland development, exocrine system development, and positive regulation of embryonic development) and cell cycle processes (negative regulation of mitotic cell cycle, regulation of cell cycle G2/M phase transition, and regulation of mitotic cell cycle); these processes are highly relevant to IUGR skeletal muscle development [6,44]. The enriched signaling pathways of differentially expressed miRNAs in the KEGG results were highly overlapping, accounting for 46.27% and 38.75% in the IUGR and normal groups, respectively (Table S3). Commonly enriched signaling pathways include endocytosis [45], TNF signaling pathway [46], autophagy-animal [47], FoxO signaling pathway [48], and MAPK signaling pathway [49], which are closely related to skeletal muscle development. The FoxO signaling pathway and MAPK signaling pathway are related to skeletal muscle development in the IUGR [44,50]. In the independently enriched signaling pathways, the high-expressed miRNAs in the normal group were enriched in the GnRH signaling pathway, PI3K-Akt signaling pathway, growth hormone synthesis, secretion and action, insulin secretion, and other pathways closely related to IUGR development [6,10]; however, the highly expressed miRNAs in the IUGR group were mainly enriched in amino acid and fatty acid metabolism pathways. Both the expression signature and the functional enrichment can effectively characterize the skeletal muscle development in normal and IUGR pigs. Our results indicate that the screened differential miRNAs have the potential to become IUGR pig skeletal muscle markers.

### 4.2. Characterization of miRNAs in Skeletal Muscle of Normal and IUGR Neonatal Piglets

Fetal development depends on the nutrient supply of the cord blood, and the miRNA in the cord blood plays an important role in fetal development [51,52]. Combined analysis of IUGR pig umbilical cord blood miRNAs data in our group found that miR-1, miR-365-3p, miR-202-5p, miR-150, miR-10b, miR-451, miR-17-5p, miR-144, miR-15a, miR-486, miR-425-5p, and miR-423-3p were co-highly expressed in normal porcine skeletal muscle and umbilical cord blood. In contrast, miR-122-5p, miR-29b, miR-29c, miR-29a, and miR-210 were highly expressed in IUGR pig skeletal muscle and umbilical cord blood. Interestingly, miR-29a, miR-29b, and miR-29c belong to the same miRNA family; these miRNAs are all related to skeletal muscle development, among which miR-1, miR-10b, miR-486, miR-122, and miR-29b have been widely reported [53,54]. The miR-29 family includes miR-29a, miR-29b, and miR-29c [55]. The IUGR animals had reduced body and organ weight, low immunity, and metabolic disorders. The reduction in birth weight and organ weight was mainly due to the reduced number of cells in the tissue. Further, the miR-29 family regulates the proliferation of normal cells [56] or cancer cells [57]. In addition, the miR-29 family is closely related to myoblast differentiation and muscle atrophy [58]. According to functional enrichment analysis, the target genes of the miR-29 family were primarily involved in protein modification (protein methylation, protein alkylation, and histone methylation) and cell differentiation (regulation of cell differentiation and endodermal cell differentiation); these processes influence skeletal muscle formation and are closely related to muscle mass [6,9]. The KEGG results showed that among the miR-29 family-enriched signaling pathways, the PI3K-Akt signaling pathway was the core pathway regulating muscle atrophy [48]. Both the expression pattern of the miR-29 family and the enrichment analysis of target gene functions suggest that the miR-29 family might play an essential role in developing skeletal muscle in IUGR animals.

CCND1, CCNB1, and CDK4 are common cell cycle genes often used as cell proliferation marker genes [59]. During early myogenesis, MYF5 partially enhances early myogenesis by coordinating increased CCND1 transcription and CCND1 mRNA translation [60]. IGF1 is an essential regulator involved in IUGR formation, and IGF1 is regulated by fetal glucose supply [61]. IGF1 has mitotic effects that induce somatic cell development and proliferation; it affects the transport of glucose and amino acids in the placenta, and IGF1 deficiency can lead to decreased fetal growth rate [6]. Further, studies have shown that human mutations in the IGF1 and IGF1 receptor genes lead to intrauterine and postpartum growth restriction [62]. The 3’UTR of IGF1 has two binding sites for the miR-29 family, and CCND1 has one binding site for the miR-29 family. According to the dual-luciferase reporter system, all three binding sites had a binding relationship. Further, in vitro experiments showed that miR-29a might regulate cell proliferation in vitro by targeting IGF1 and CCND1 and promoting the atrophy of mature myotubes. Co-transfection experiments with porcine cord blood exosomes suggested that the miR-29 family might be the core molecules involved in regulating fetal skeletal muscle development via cord blood miRNAs.

## 5. Conclusions

**Figure 8:**
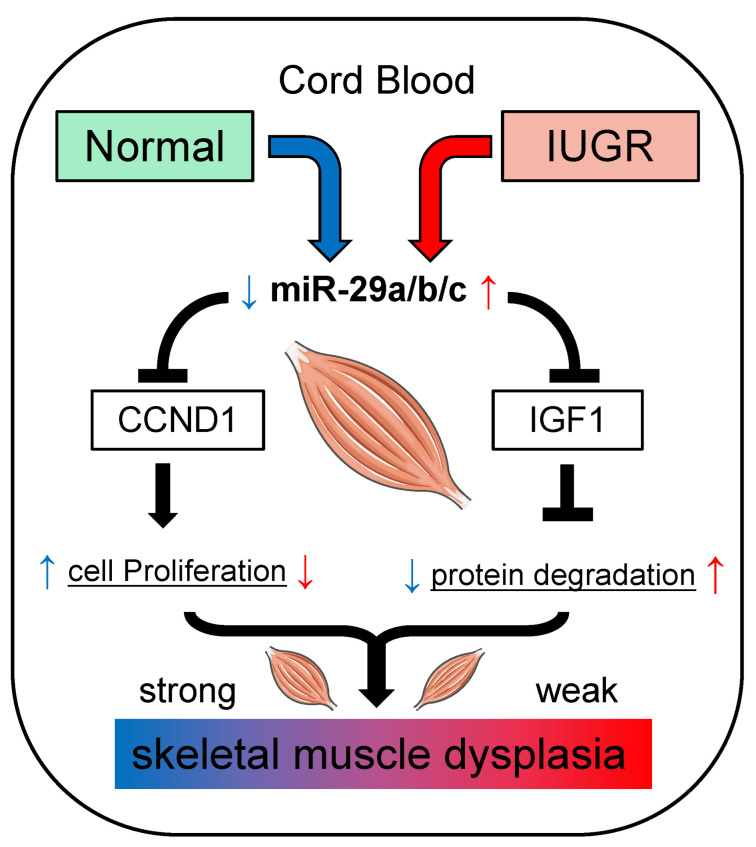
The miR-29 family is involved in the regulation of skeletal muscle development in IUGR pigs. This section may be divided into subheadings; it should provide a concise and precise description of the experimental results, their interpretation, as well as the experimental conclusions that can be drawn.

In conclusion, our study reported the expression patterns and characteristics of miRNAs in normal and IUGR neonatal piglet skeletal muscle and found that myogenic miRNAs were reduced in IUGR piglet skeletal muscle. In addition, the combined analysis of umbilical cord blood miRNAs data indicated that the miR-29 family might be the core regulatory molecules involved in IUGR pig skeletal muscle dysplasia. The miR-29 family is involved in cell proliferation, differentiation, and muscle atrophy by targeting CCND1 and IGF1 (Figure 8); these results increase the understanding of the effects of umbilical cord blood miRNAs on fetal skeletal muscle development and provide a reference for studying skeletal muscle development in IUGR animals.

## Data Availability

The raw reads produced in this study were deposited in the NCBI Sequence Read Archive (SRA), the records can be accessed by accession number PRJNA816268. The remaining data that support the findings of this study are available from the corresponding author upon reasonable request.

## Supplementary Materials

The following supporting information can be downloaded at: https://www.mdpi.com/article/10.3390/biom12091193/s1. Table S1: Sequences of PCR primers used in this study; Table S2: GO and KEGG pathway enrichment analysis of differential miRNAs; Table S3: The miRNAs in a Venn diagram; Table S4: The quality of RNA.
